# Regenerative treatment of canine osteogenic lesions with Platelet-Rich Plasma and hydroxyapatite: a case report

**DOI:** 10.3389/fvets.2024.1459714

**Published:** 2024-09-23

**Authors:** Katia Barbaro, Giorgio Marconi, Elisa Innocenzi, Annalisa Altigeri, Alessia Zepparoni, Valentina Monteleone, Cristian Alimonti, Daniele Marcoccia, Paola Ghisellini, Cristina Rando, Stefano Ottoboni, Julietta V. Rau, Roberto Eggenhöffner, Maria Teresa Scicluna

**Affiliations:** ^1^Istituto Zooprofilattico Sperimentale del Lazio e della Toscana “M. Aleandri”, Rome, Italy; ^2^Centro Veterinario San Tommaso, Rome, Italy; ^3^Department of Surgical Sciences and Integrated Diagnostics (DISC), Genova University, Genoa, Italy; ^4^Istituto Struttura della Materia, Consiglio Nazionale delle Ricerche (ISM-CNR), Rome, Italy

**Keywords:** case report, platelet-rich plasma, hydroxyapatite nanoparticles, veterinary regenerative medicine, ImageJ analysis

## Abstract

**Introduction:**

This study examined the efficacy of a therapy based on a combination of Platelet Rich Plasma and hydroxyapatite nanoparticles in a severe clinical case involving a young Rottweiler with a complex spiral fracture of the tibia.

**Method:**

Following a worsening of the lesion after traditional surgical intervention, the subject was treated with the combined therapy. X-rays were taken at the following stages: immediately post-surgery, four weeks post-surgery, and 10 days post-treatment. Fracture gap and callus density measurements were obtained using ImageJ analysis, allowing for a detailed quantitative assessment of bone regeneration over time.

**Results:**

Post-operative radiographs indicated a clinical worsening of the fracture, revealing an increased fracture gap due to bone loss. However, significant improvements were observed ten days following the treatment, with a marked reduction in fracture gaps and increased callus density. These results demonstrated a notable acceleration in bone healing and callus formation compared to typical recovery times for similar lesions.

**Conclusion:**

The method showed potential for enhancing osteogenic regeneration, facilitating faster healing of serious orthopedic injuries compared to traditional methods.

## 1 Introduction

In veterinary medicine, healing osteogenic lesions in dogs presents significant challenges. When conventional treatments proved insufficient, there is a potential need for limb amputation, necessitating more effective therapeutic approaches. The use of hemocomponents as Platelet-Rich Plasma (PRP) (1), or the application of mesenchymal stromal cells (2), represented a pivotal strategy for tissue repair and regeneration. This study addressed an urgent osteogenic lesion in a canine patient by introducing a novel therapeutic approach that combines the synergistic potential of PRP and hydroxyapatite nanoparticles (HAp).

PRP, rich of growth factors, played a crucial role in driving the repair processes, including neoangiogenesis, collagen deposition, and the recruitment of stem cell to the injury site (1, 3). In regenerative medicine, PRP demonstrated versatility and efficacy in facilitating the proliferation and differentiation of cells within the injured area, thereby promoting rapid healing across a range of injuries (4). The regenerative capabilities of PRP were further enhanced when used in anti-aging and tissue regeneration strategies, showcasing its adaptability and potency across diverse applications (5, 6). Compared to more complex procedures such as those involving mesenchymal stromal cells, PRP’s simplicity in preparation and application enhanced its appeal in clinical and scientific settings (7).

In veterinary clinical practice, PRP-based therapeutic strategies were suggested for various orthopedic pathologies across different species to promote tissue repair and regeneration (8). The objective of the present work was to investigate recovery facilitated by the combined action of PRP and HAp in a clinical case. HAp was chosen for its well-known osteoconductive properties, providing essential support for tissue regeneration. It functioned as a distributed aggregation center rather than a scaffold, promoting cell adhesion, proliferation, and differentiation, thereby augmenting the regenerative potential of PRP (9). The synergic action of PRP and HAp offered a reliable approach to osteogenic regeneration, representing a viable fundamental support to conventional surgical interventions.

## 2 Case description and methods

### 2.1 Clinical case

A clinical case study was conducted on a 13-month-old male Rottweiler with a spirally fractured tibia. Spiral fractures, typically caused by intense twisting forces, are characterized by a helical break around the bone. In the case of our subject, the fracture presented a significant challenge due to the complex nature of the injury. The initial treatment involved conventional orthopedic surgical procedures, which included the insertion of plates for limb stabilization, as is frequently done in these situations to facilitate alignment and accelerate the healing process. However, in this case, the post-operative period was marked by substantial resorption of the bone tissue. The necrotization of preexisting bone tissue led to a significant decrease in bone mass at the fracture site. The patient experienced extreme pain due to this extensive bone resorption, underscoring the severity of the condition and the complexity of the recovery process.

Given these circumstances, limb amputation was deemed inevitable. However, before proceeding with the amputation, the owner was informed of an alternative procedure that deviated from conventional treatment methods and promised a potentially more favorable outcome of the subject. The owner was fully informed about the potential benefits and risks of the procedure and subsequently provided written consent, allowing for the implementation of the new protocol, which was developed to address the shortcomings of the conventional, unsuccessful surgical treatment. Improved quality of life, accelerated healing, pain management, and prevention of bone resorption were all goals of the protocol.

### 2.2 Preparation of Platelet-Rich Plasma

**Figure 1:**
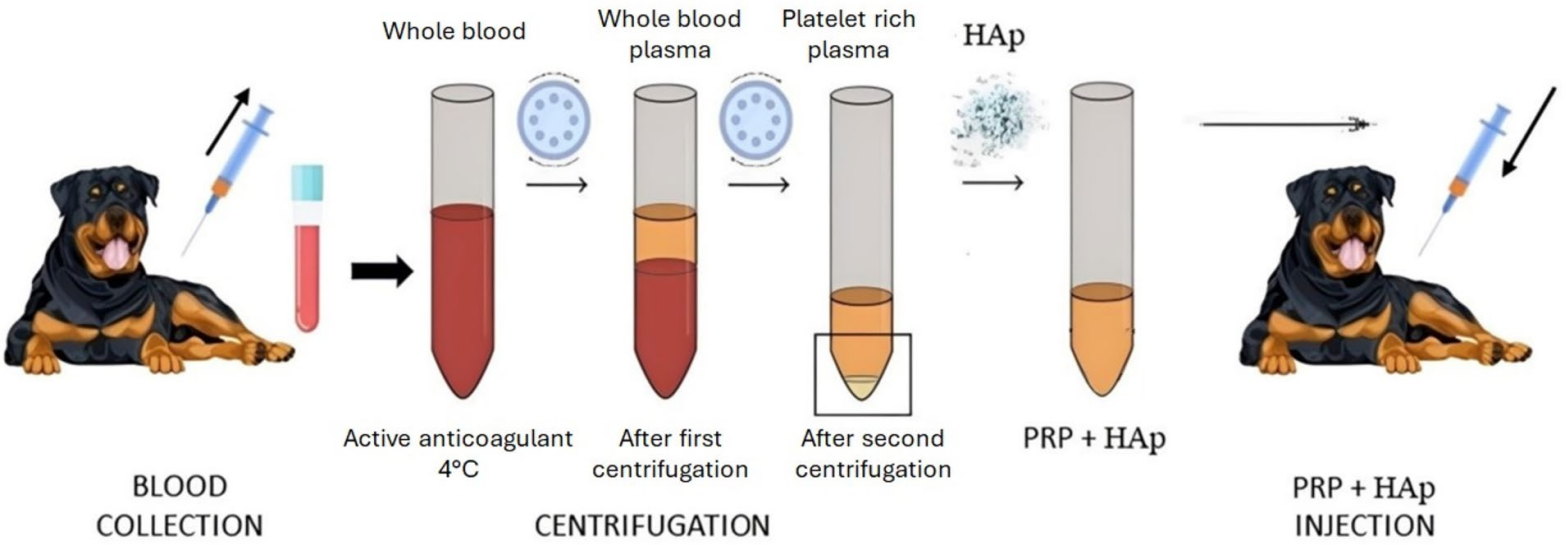
Scheme of the therapeutic approach: blood collection, PRP + HAp therapeutic preparation and inoculation.

Autologous PRP was prepared by drawing approximately 50 mL of peripheral blood under sterile conditions into a test tube containing sodium citrate (BD Vacutainer®). The tubes were centrifuged at 350 *g* for 30 min to separate the blood into distinct phases. The plasma layer above the buffy coat, which contains the platelets and leukocytes, was then extracted, and subjected to a second centrifugation at 900 *g* for 15 min. The resulting platelet pellet was resuspended in 10 mL of plasma. In this study, the presence of leukocytes was retained in the PRP preparation, granting their dual role in enhancing tissue healing and counteracting potential microbial contamination in bone tissue (10). All procedures were conducted under sterile conditions within a class II biosafety cabinet. Figure 1 illustrates three vials post-centrifugation: one containing whole blood, one with whole blood plasma, and one with PRP.

### 2.3 Platelet-Rich Plasma and hydroxyapatite inoculation

Three cubic centimeters of powdered HAp (ENGIpore, Finceramica, Italy) and 5% calcium chloride (CaCl_2_ 10%) were incorporated into 10 mL of autologous PRP to stimulate the platelets to release growth factors (11). The inoculated resulting composite was five times more concentrated than the collected sample. The mineral calcium apatite, or HAp, is a naturally occurring form predominantly found in tooth enamel and bone. The osteoconductivity, bioactivity and biocompatibility of this material make it a suitable choice in bone tissue engineering. One of its main features is its trabecular structure, which resembles natural bone and ideal for applications involving the healing of bone tissue. The high porosity of the material facilitates the rapid absorption of biological fluids, allowing for rapid cellular colonization of the fractured area, which is fundamental for effective bone regeneration. Furthermore, ENGIpore is produced with the highest quality standards and poses no risk of viral transmission, making it a safe choice for clinical applications (12). The final step in the present procedure involved administering a composite of PRP and HAp to the canine patient, specifically designed and executed to promote tissue healing and regeneration. The inoculation was performed percutaneously following the indications of the radiographic images to locate the lesion. The needle was inserted behind the plate and the composite was delivered directly into the fracture site under sedation and with the maximum sterility.

### 2.4 Clinical and radiographic follow-up

**Figure 2:**
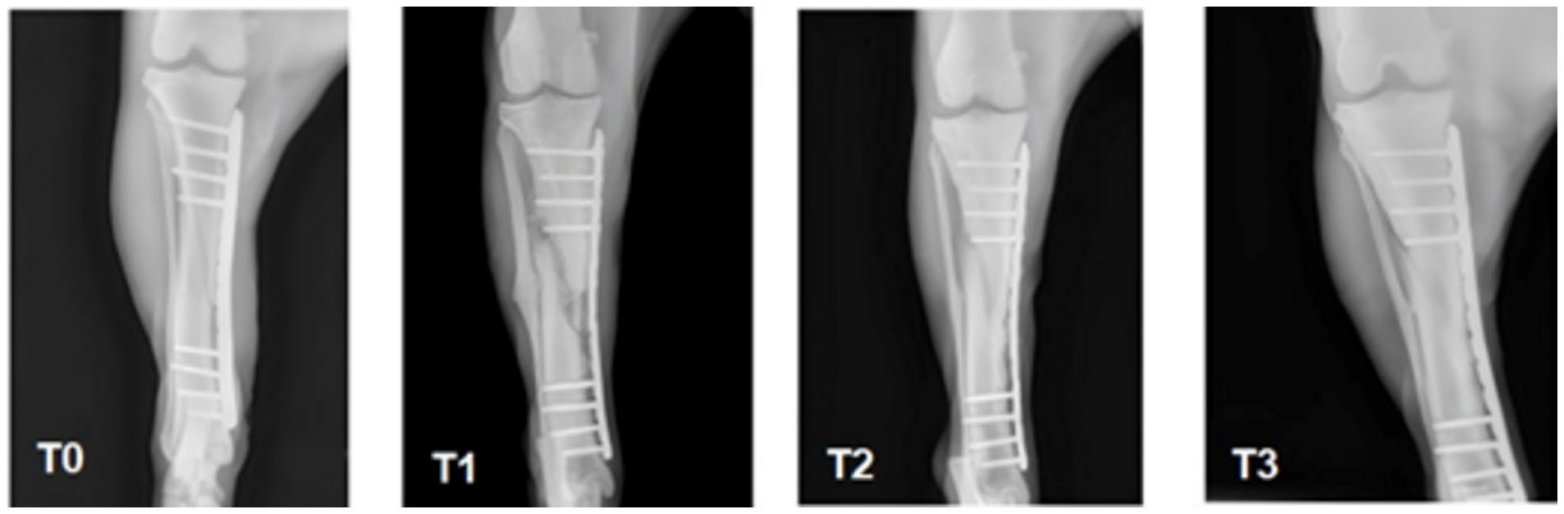
X-rays follow up of the dog’s tibia at different timings. T0, post-surgery; T1, at 4 weeks after surgery; T2, at 10 days after treatment with PRP and HAp; T3, proof of recovery after 8 weeks.

The dog’s clinical status and physical activity were monitored weekly. Radiographic examinations were conducted following the initial surgery, four weeks post-surgery, and at ten days and two months after the administration of autologous PRP and HAp. Specifically, the fracture and the subsequent surgery, including the application of the medial plate (Figure 2-T0) were documented. After four weeks radiographic control showed significant bone tissue absorption, leading to the inoculation of PRP with HAp (Figure 2-T1). Ten days after inoculation, radiographic control revealed significant consolidation of the bone callus (Figure 2-T2). Eight weeks post-inoculation, further radiographic control confirmed additional fracture consolidation and functional recovery, as evidenced by increased limb musculature (Figure 2-T3). After two months, the animal’s recovery was evident, and the veterinarian determined that further sedation and X-ray exposure were unnecessary.

### 2.5 Image analysis of the fracture healing using ImageJ

X-ray images of the fracture, taken before and after treatment, were analyzed using ImageJ software (RRID: SCR_003070) to specifically determine fracture gap lengths and optical intensity (version 1.54j, National Institutes of Health, Bethesda, MD, USA). The images were first aligned and made superimposable to enable a comparable view. Initially, the images were superimposed onto a reference grid before fine-tuning the alignment based on anatomical landmarks visible in the X-rays. In ImageJ, threshold settings were then adjusted to ensure consistent brightness and contrast values across the reference grid area. These standardizations were essential for accurately measuring characteristic distances before and after the combined treatment with PRP and HAp. A rectangular region encompassing the fracture site was specifically selected, ensuring similar pixel dimensions for both pre-and post-treatment images. The intensity values within this region were measured in arbitrary units, maintaining consistency across both sets of images, checking that these values were not significantly affected by the sizes of the selected regions.

## 3 Results

The synergistic combination of blood components and biomaterials with appropriate physicochemical properties significantly enhances tissue regeneration and accelerates healing. In the case study illustrated in Figure 2, the effectiveness of this approach is demonstrated. Initially, the canine patient underwent a standard surgical procedure for the implantation of a medial plate. Figure 2-T0 shows the radiograph taken post-surgery for tibial stabilization, providing a baseline before any additional therapies were administered. This first X-ray image serves as a benchmark for assessing the efficacy of the subsequent treatment.

Figure 2-T1 taken four weeks post-surgery, reveals a worsening of the lesion characterized by bone substance loss. This indicates that while the medial plate was necessary for stabilizing the fracture, it was insufficient to promote healing on its own. The visible bone tissue absorption highlights significant challenges in the healing process, possibly due to the body’s adverse reaction to the implanted material or the severity of the initial injury. The lesion’s deterioration even after surgery underscores the inadequacy of traditional surgical methods in this case, emphasizing the critical need for novel therapies like the one explored in this study with PRP and HAp.

The turning points in the healing process was the introduction of the treatment that combined HAp nanoparticles with PRP. As shown in Figure 2-T2, this treatment led to significant improvements, clearly demonstrating its effectiveness. The T2 X-ray is particularly noteworthy as it reveals the emergence of a bone callus, a definitive sign of osteogenic regeneration, as quantified in the subsequent analysis that compares these two critical images. Even a visual comparison suggests that the treatment promoted neoangiogenesis and osteoinduction, suggesting that the body’s natural bone healing processes were stimulated. Additionally, the patient demonstrated a robust functional response, evidenced by increased use of local muscles, indicating both recovery and the restoration of limb functionality.

Figure 2-T3, representing the final stage of recovery after eight weeks, shows continued improvement in the lesion’s condition following further treatment with PRP and HAp. This final X-ray confirms the long-term efficacy of the treatment, demonstrating increased limb musculature in addition to bone consolidation, i.e., indicating functional restoration.

Collectively, Figure 2 T0÷T3 illustrates the patient’s therapeutic pathway from the initial condition through unsuccessful surgical intervention to successful recovery following the innovative PRP and HAp treatment. The sequence of images describes the effectiveness of the treatment and clarifies the potential of incorporating hemocomponents and biomaterials into regenerative therapies.

**Figure 3:**
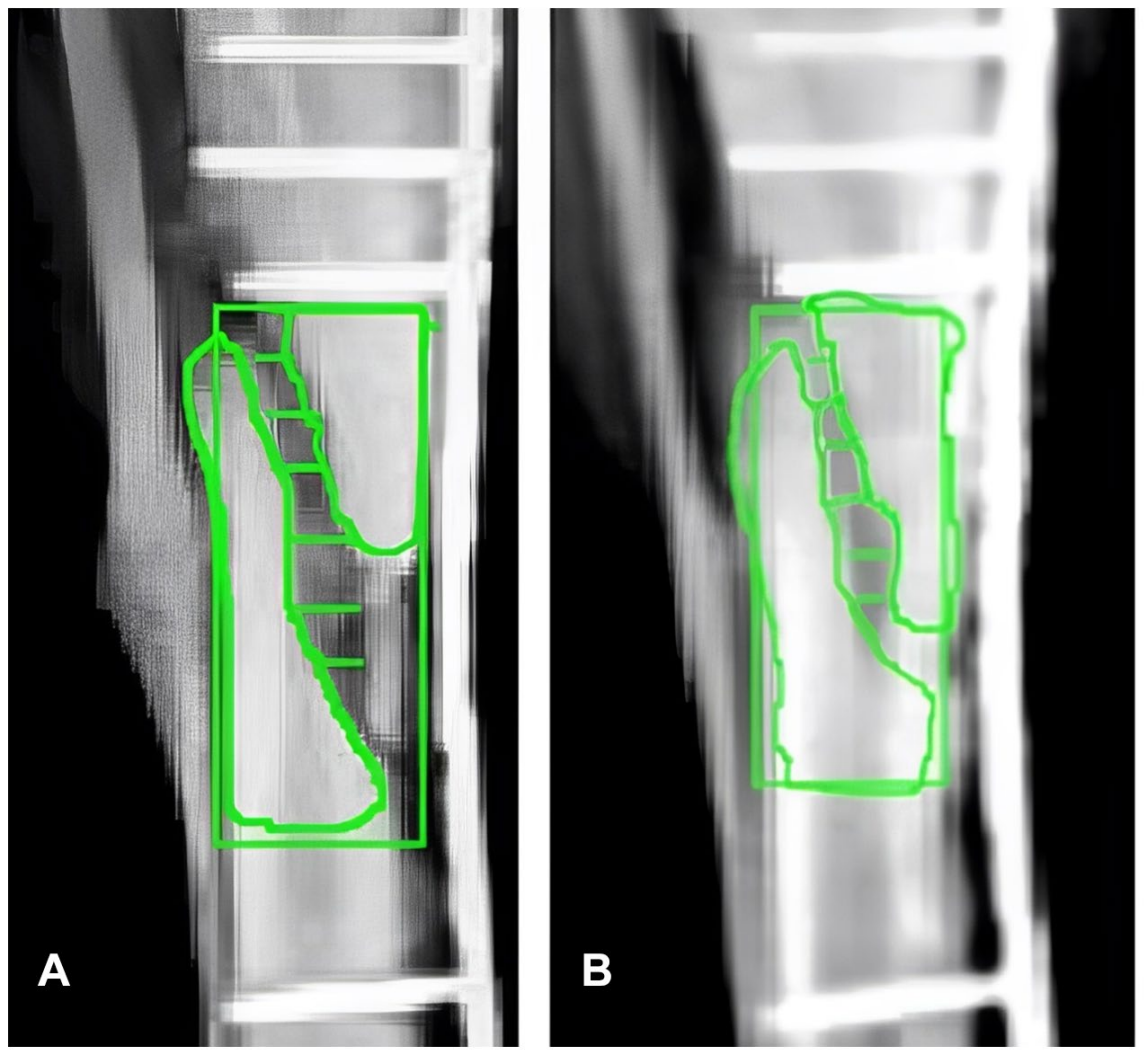
Comparative X-ray analysis and ROI quantification using ImageJ. **(A)** X-ray image of the canine tibial fracture four weeks post-surgery, before treatment with PRP and Hap. **(B)** X-ray image ten days post-treatment with PRP and Hap. Six green segments (S1–S6) indicate significant positions along the fracture used for measuring the fracture gap. Two regions of interest (ROIs) above and below the fracture, along with a rectangular area encompassing the fracture site are highlighted for intensity analysis. The quantitative data derived from these ROIs, are presented in Table 1.

The most significant comparison, essential for demonstrating the challenges in the healing process, is between the condition four weeks post-surgery (Figure 3A) and ten days after PRP and HAp treatment (Figure 3B). Both Figures 3A,B were obtained by matching the above Figures 2-T1 and T2, respectively, in both the positions of fixed elements and intensities. The results of the ImageJ analysis are presented in Table 1, where six green segments are placed at key points along the fracture, and two areas, above and below the fracture, are highlighted within the rectangle representing the regions of interest (ROIs) in both images. The length of these segments indicates the extent of the fracture, while the ROIs are used to assess bone callus formation above and below the fracture site.

**Table 1:** Quantitative analysis of fracture healing and callus density post-treatment.

Segment/area	Pre-treatment	Post-treatment
S1	0.24	0.09
S2	0.30	0.16
S3	0.15	0.11
S4	0.37	0.11
S5	0.35	0.23
S6	0.13	0.12
A1 (Fracture Site)	169	206
A2 (Above Fracture)	203	207
A3 (Below Fracture)	206	210

The changes in fracture segment lengths (S1–S6) and intensity values (A1, A2, A3) before and after the treatment with PRP and HAp are reported. The significant reduction in segment lengths and increased intensity values indicate enhanced bone healing and callus formation in the conditions of post-treatment. The length and intensity values, measured in arbitrary units, were consistent in both the pre-treatment and post-treatment images, allowing for direct comparison of the changes observed.

The numerical data shown in Table 1 include the segment lengths (S1–S6), numbered from top to bottom, and three intensity values (A1, A2, A3). As shown in Figure 3, three significant areas were identified to detect brightness correlated with bone regrowth in the fractured region (indicated with symbols A1, A2, and A3 in Table 1). A1 represents the rectangular area surrounding the fracture, A2 is the area above the fracture, and A3 is the area below the fracture. These regions were precisely positioned and sized for a comparative analysis of bone regeneration. Quantitative information on callus density pre-and post-treatment was obtained by assessing the intensity within these ROIs. Reliable and repeatable results in our analysis of tibial fracture healing were ensured by standardizing these regions and measurement protocols.

## 4 Discussion

The transition from experimental research to clinical practice in PRP and HAp-based bone regeneration marks a pivotal advancement in regenerative therapies. Bridging experimental research and clinical practice in PRP and HAp-based bone regeneration was facilitated by crucial *in vitro* studies that initially demonstrated the significant potential of PRP and HAp in bone regeneration (13). The findings from these studies have informed and refined procedures that are now being applied in clinical practice (12, 14–16).

The data presented in Table 1 and Figures 2, 3 illustrate remarkable improvements in fracture healing post-treatment with PRP and HAp. Specifically, the fracture segments (S1–S6) exhibited significant reductions, with the most substantial decrease observed in the central region of the fracture. This indicates effective narrowing of the fracture gap, a critical marker of bone regeneration. Furthermore, the intensity values representing callus density in the X-ray images also showed notable increases. The intensity at the fracture site (A1) increased from 169 to 206 a.u., indicating denser bone formation. Similarly, the regions above (A2) and below (A3) the fracture showed slight increases in intensity values from 203 to 207 a.u. and from 206 to 210 a.u., respectively. These changes suggest that the combined PRP and HAp therapy significantly enhanced callus formation and overall bone healing.

The results of the present investigation are compared with findings in the literature, as summarized in Table 2, to support our conclusions and explore different outcomes.

**Table 2:** Detailed overview of *in vivo* studies on PRP and HAp for bone regeneration, including outcome analysis and relevance to the current clinical study, organized chronologically from the earliest to the most recent publications.

Study	Species	Defect type	PRP/HAp application	Outcome	Comments
Kon et al., 2010 (20)	Sheep	Osteochondral	Collagen-HAp scaffold + PRP	PRP decreased the osteochondral regeneration capability of the scaffold	The combination of PRP and scaffold led to highly amorphous cartilaginous repair tissue underlying bone tissue.
Yamada et al., 2010 (14)	Dog	Bone defects—dental implant	PRP + Dental pulp stem cells	Promoted bone regeneration	Shows the potential of PRP in combination with stem cells for enhanced bone healing.
Knapen et al., 2013 (21)	Rabbits	Bone regeneration	L-PRF (a variant of PRP)	No significant effect on bone regeneration	Contrasts with this study’s positive outcome, suggesting that different PRP formulations may yield varied results.
Zhang et al., 2020 (17)	Rats	Critical-size calvarial defects	PRP + ADSC and HAp	Enhanced bone formation and defect closure	Demonstrates synergistic effects of PRP and HAp on bone healing in critical-size defects primarily treated with ADSC
Jiang et al., 2021 (18)	Rabbits	Osteochondral	3D-printed PRP-GelMA hydrogel	Promoted osteochondral regeneration through M2 macrophage polarization	Enhanced migration, proliferation, and differentiation of BMSCs, and improved cartilage and subchondral bone regeneration.
Henkel et al., 2021 (22)	Sheep	Large volume tibial segmental defects	mPCL-TCP scaffolds with PRP and rhBMP-7	Promoted bone regeneration at both short-term and long-term time points	Large volume tibial defects in sheep are treated with varying degrees of success in bone regeneration and mechanical integration.
Lee et al., 2018 (16)	*In vitro*	Bone regeneration	PRP + HAp scaffold (Ca-deficient)	Enhanced bone regeneration	Demonstrates the effectiveness of PRP combined with Ca-deficient HAp to favor osteoblast proliferation.
Venkataiah et al., 2021 (25)	Various mammals	Bone defects	Cell-scaffold constructs with PRP	Promoted bone regeneration	This work further supports the clinical applicability.
Rapone et al., 2022 (23)	Humans	Maxillary sinus augmentation	Algipore (HAp) and PRP	Predictable long-term results; comparable to Bio-Oss without PRP	The potential of PRP in clinical settings is highlighted, although focused on a different bone lesion type.
Inchingolo et al., 2022 (24)	Various	Bone defects regeneration	Engineered grafts with PRP	Enhanced bone regeneration	Reinforces the concept of PRP-enhanced grafts for bone healing akin to this study’s use of HAp.
Sharun et al., 2023 (19)	Rabbits	Atrophic non-union fractures	PRP and AdSVF	Improved healing in orthopedic conditions	Supports this study’s findings by demonstrating PRP’s effectiveness in a different animal model.

The potential of PRP in promoting bone regeneration has been assessed in various studies. For example, Yamada et al. (14) demonstrated the effectiveness of PRP in combination with dental pulp stem cells across various species. Their findings showed a significant promotion of bone healing, suggesting that PRP could synergize with stem cells to enhance the regenerative process. This study underscores the role of PRP in facilitating stem cell-mediated bone repair, which aligns with our findings where PRP contributed to enhanced bone regeneration in a clinical setting.

Similarly, Zhang et al. (17) explored the application of PRP in conjunction with HAp for treating critical-size calvarial defects in rats. The study reported enhanced bone formation and defect closure, demonstrating the synergistic effects of PRP and HAp. This supports the hypothesis that PRP can significantly improve the osteoconductive properties of HAp, accelerating the bone healing process, particularly in challenging defect scenarios. These findings (17) resonate with the outcomes observed in our study, where the combination of PRP and HAp led to marked improvements in bone regeneration.

Additionally, Jiang et al. (18) utilized a 3D-printed PRP-GelMA hydrogel in a rabbit model to promote osteochondral regeneration. The study highlighted that this combination successfully induced M2 macrophage polarization, leading to enhanced migration, proliferation, and differentiation of bone marrow stem cells (BMSCs). These findings provide compelling evidence that PRP, when used with advanced scaffold technologies, can significantly boost cartilage and subchondral bone regeneration.

Sharun et al. (19) further supported the effectiveness of PRP in large defect models, showing that it significantly enhanced bone healing in rabbit orthopedic conditions, particularly when combined with stromal vascular fraction (SVF). These findings align with our results and contribute to the growing body of evidence supporting the clinical utility of PRP in bone regeneration, especially in conjunction with SVF.

Despite these promising results, some studies have reported inconsistent or less favorable outcomes. For example, Kon et al. (20) found that PRP, when combined with collagen-HAp scaffolds, actually decreased the osteochondral regeneration capability in sheep models. This study suggested that PRP might not always enhance regenerative outcomes and, in certain combinations, could interfere with the healing process. This finding emphasizes the need for careful consideration of the materials and conditions under which PRP is applied.

Knapen et al. (21) also reported no significant effects on bone regeneration when using leukocyte-and platelet-rich fibrin (L-PRF), a variant of PRP, in a rabbit model. This study indicated the variability in PRP formulations and their effects, suggesting that not all PRP preparations are equally effective. These results indicate also that the specific formulation and application method of PRP are crucial for its success in promoting bone healing.

Similarly, Lee et al. (16) investigated the combination of PRP with a calcium-deficient hydroxyapatite (Ca-deficient HAp) scaffold for bone regeneration. The study found that PRP, when used with this specific type of HAp scaffold, enhanced osteoblast proliferation and bone regeneration. Thus, PRP is observed to improve bone healing, though the effects can vary depending on the specific formulation.

Several studies have raised concerns related to the translation of PRP benefits across different models and clinical settings. Henkel et al. (22) established a new preclinical model for large-volume tibial defects in sheep and demonstrated the potential of PRP combined with synthetic scaffolds and growth factors. However, the varying degrees of success observed in the study underscore the challenges in standardizing PRP applications across different models, emphasizing the need for customized approaches in diverse clinical scenarios.

Rapone et al. (23) compared the effectiveness of PRP combined with Algipore (HAp) in maxillary sinus augmentation to that of Bio-Oss without PRP in human subjects. Their findings showed that PRP could achieve predictable long-term outcomes, comparable to those of existing treatments, though not necessarily superior. This study illustrates the nuanced role of PRP in clinical applications, where its benefits may depend on specific clinical contexts and conditions.

Inchingolo et al. (24) and Venkataiah et al. (25) both underlined the variability in outcomes when using PRP in combination with other regenerative agents. Variable effects on bone regeneration in alveolar bone defects were reported (24), emphasizing the need for standardized PRP preparation protocols. Enhanced bone healing in canine mandibular defects with PRP and HAp combined with autologous stem cells were observed (25), but the results were inconsistent, pointing to the complexity of PRP applications.

## 5 Conclusion

The treatment of a complicated tibial fracture of a canine patient with HAp and PRP was effective, as indicated by increased bone density and reduced fracture gaps confirmed by X-ray analysis. The minimally invasive technique led to effective bone healing and functional recovery representing thus a potential alternative to conventional surgical methods in similar cases. Our results validate the role of PRP in bone regeneration and the osteoconductive properties of HAp, though careful PRP formulation is recommended due to the variability in outcomes observed in other studies. Further research is needed to improve the understanding of the interactions between PRP and HAp and their potential applications in human medicine.

## Data Availability

The raw data supporting the conclusions of this article will be made available by the authors, without undue reservation.
